# Development of Corona-virus-disease-19 Vaccines

**DOI:** 10.31662/jmaj.2021-0068

**Published:** 2021-07-09

**Authors:** Hideki Hasegawa

**Affiliations:** 1 Center for Influenza and Respiratory Virus Research, National Institute of Infectious Diseases, Tokyo, Japan

**Keywords:** COVID-19, SARS-CoV-2, vaccine, VADE, animal model

## Abstract

The year 2020 opened with news of an epidemic of pneumonia caused by a new coronavirus similar to the SARS coronavirus in Wuhan, China, and subsequently caused a worldwide pandemic. In Japan, the first infected person was found in January, and later, more than 700 passengers and crew members of the *Diamond Princess*, a large cruise ship that called at Yokohama port, were found to be infected, and the ship was forced to respond to the outbreak. The causative virus was quickly identified as a beta coronavirus similar to the severe acute respiratory syndrome (SARS) coronavirus of 2003 and was named SARS coronavirus 2 (SARS-CoV-2). The disease was named COVID-19. SARS-CoV-2, like SARS-CoV and MERS-CoV, infects the epithelial cells of the lungs and causes viral pneumonia. As of March 7, 2021, more than 116 million people have been infected and more than 2.5 million people have died worldwide. As a result of the global pandemic, regional blockades have been imposed around the world, and the development of vaccines and therapeutic agents has become an urgent necessity in order to restore normal social activities. In this review, the experience of the development of SARS-CoV-2 vaccine is described.

## SARS and MERS Vaccine Development

The model for starting vaccine development was the strategy of vaccines for SARS and MERS, which are similar pneumonia diseases caused by beta coronaviruses similar to COVID-19 ^(1), (2)^ caused by SARS-CoV-2. However, the reality is that there is no approved vaccine for SARS or MERS yet, and there are issues that must be overcome.

In the case of the SARS-CoV vaccine, attempts were made to develop recombinant vaccines based on the S protein, the surface protein of the virus, attenuated live vaccines, inactivated whole-particle vaccines, and vector vaccines ^(3)^. However, these vaccines were not able to induce sterilized immunity in animal models. In addition, incomplete immunity may cause vaccine-associated disease exacerbation (VADE) such as exacerbation of pneumonia and eosinophilic infiltration due to an infection after vaccination, indicating the need for careful attention to vaccine safety. In many cases, S-protein-based vaccines have been shown to reduce post-infection viral titers and improve survival, but similar VADEs have been observed in the development of MERS-CoV vaccines ^(4), (5)^. In the development of a vaccine for SARS-CoV-2, special attention must be paid to ensure safety in humans. Another factor to be considered in the development of a vaccine for SARS-CoV-2 is the characteristics of immunity induced by human coronavirus infection. Although some viral infections confer lifelong immunity, studies of antibody responses in SARS- and MERS-infected patients have shown that the immunity induced by human coronavirus infection does not last for a long time but declines within a few years ^(6), (7)^. The same may be true for the immune response to SARS-CoV-2. A similar phenomenon has been reported in the response of SARS-CoV-2, that is, the repeated infection by another virus within a short period of time after recovery. Although it is unclear whether the immune response to SARS-CoV-2 induced by a vaccine is necessarily the same, it is important to note the duration of immunity. Thus, the knowledge gained from the SARS and MERS vaccine studies will be very important in the development of a vaccine against SARS-CoV-2.

## Vaccine Platforms

**Figure 1.**
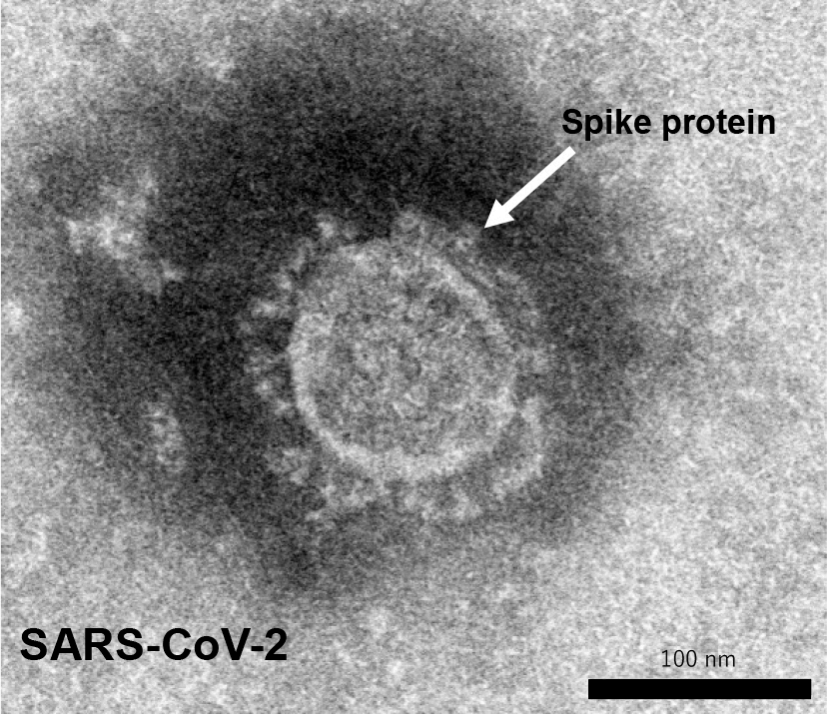
Transmission electron micrograph of SARS-CoV-2. The white arrow points to the spike protein.

The development of a vaccine against SARS-CoV-2 is currently being pursued at a rapid pace around the world using various platforms and the full power of the latest technologies (Table 1). Some of the vaccines have already been commercialized and approved in many countries. Among the many platforms, messenger RNA (mRNA) vaccines, which are genetically engineered and take less time to manufacture, have been developed. They have successfully finished phase III clinical trials with 95% vaccine efficacy ^(8)^ and have been approved in many countries. Unlike conventional vaccines that use viral components as antigens and induce an immune response, these vaccines use mRNA encoded by the virus and inoculate the vaccine with the mRNA. The genome sequence of SARS-CoV-2 has been rapidly sequenced and published by Chinese researchers ^(2), (9), (10)^. Vaccine studies for SARS and MERS have suggested that antibodies targeting the spike protein (S protein) on the virus surface (Figure 1) are useful in preventing infection by inhibiting the binding of the virus to its receptor, ACE2. In addition to vaccines that directly utilize genes, research institutes and manufacturers in Europe and the United States have been developing vaccines that express S protein using viral vectors. Oxford University has developed a vector vaccine based on chimpanzee adenovirus vectors that has also completed phase III clinical trials successfully with high vaccine efficacy ^(11)^ and has been approved in many countries. These vaccine developments are based on new technologies that have never been used before in general in human vaccines.

**Table 1.** Vaccine Types.

Vaccine type	Advantages	Disadvantages	Examples of other vaccines
Virus vector	Easy to develop, theoretically safe	Poor precedent	Ebola, gene therapy
Nucleic acids (DNA, mRNA)	Easy to develop, theoretically safe	Poor precedent	None
Virus (inactivated or live vaccine)	Most proven	Possibility of antibody-dependent disease enhancement	Measles, rubella, influenza, and many other diseases
Recombinant protein	Proven	Adjuvant is often required	Hib, hepatitis B, HPV, pneumococcus, meningococcus

https://www.niaid.nih.gov/research/vaccine-types

When we look at the development of vaccines using conventional vaccine production, the first thing that comes to mind is an inactivated vaccine in which the virus is multiplied and inactivated for the vaccine antigen. A vaccine manufacturer in China has already finished clinical trials, with the vaccine approved for use in China. In Japan, we are cooperating with a vaccine manufacturer to develop a whole-particle inactivated vaccine. When SARS-CoV-2 was first isolated, it was thought that it would be difficult to produce inactivated vaccine due to the poor replication of the virus. However, repeated cultivation has increased the multiplication efficiency, and the yield as a vaccine antigen has improved. This inactivated vaccine is about to begin the phase I/II clinical trial in Japan.

Recombinant protein vaccines that use protein antigens are also promising candidates among the conventional vaccine platforms. We began investigating recombinant protein vaccines using the spike protein of SARS-CoV-2 as a vaccine platform at the beginning of the pandemic. This is because the technology has already been used in human vaccines and we felt confident in its safety, and also because there is a plant and technology in Japan that can produce vaccine antigens in large quantities. As the biosafety level for SARS-CoV-2 is BSL-3, it requires a special facility to replicate the virus. Therefore, it would be advantageous to produce large quantities of vaccine antigens in a short period of time without the restriction of the biosafety level of the target virus. The spike protein on the surface of SARS-CoV-2 is the key molecule to bind to the cellular receptor (Figure 1). The S protein binds to the biological receptor, angiotensin converting enzyme 2 (ACE2), and causes membrane fusion to establish the infection. Since the S protein contains a receptor binding site, it can be used as a vaccine antigen for infection prevention. To prevent this process, it is desirable to develop a vaccine that can induce antibodies that bind to the receptor binding site of the S protein and inhibit viral infection. The efficacy of the vaccine was confirmed in animal experiments, and the phase I/II clinical trials have begun at the end of 2020.

## Animal Models for Vaccine Evaluation

Another key issue in the development of a vaccine for SARS-CoV-2 is the use of proper animal models to test the efficacy and safety of the vaccine. Mice are the most popular animal to test the immune responses induced by a vaccine and to examine protection against the infection. It is also used for the safety test when infection experiments can be performed in mice. However, it has been pointed out since the discovery of the virus that SARS-CoV-2 is not infectious in wild-type mice. Therefore, it was necessary for researchers to first establish an animal model for infection. It has been reported that hamsters are susceptible to SARS-CoV-2 and can be used as an animal model for infection ^(12)^ at a very early time after the pandemic. In addition, other animals such as Ace2 transgenic mice, crab-eating monkeys, ferrets, cats, and old mice were tested for infection and immune responses.

## Vaccine-associated Disease Enhancement

In SARS vaccine research, vaccine-associated disease enhancement (VADE) has become a problem. In the mouse model, eosinophilic infiltration around blood vessels in the lungs was found in the vaccinated and infected animals, which exacerbated the pneumonia. This condition was found to depend on the type of immunity induced by the vaccine ^(10)^. At this point, it is not clear whether vaccine development against SARS-CoV-2 will result in an exacerbation of the disease. Although it is still unclear whether or not there will be an exacerbation in SARS-CoV-2 vaccine, it has been recognized internationally as an important point to be considered in vaccine development since the early stage.

In a study of the SARS vaccine, the eosinophilic infiltration observed in post-vaccination viral challenge infections was alleviated by the use of Toll like receptor agonists adjuvant ^(13)^. Since the immunity induced by different types of adjuvants differs, it is necessary to consider the effect on VADEs when using adjuvants.

## Conclusion

The efficacy of the vaccine needs to be proven in the field through large-scale clinical trials, and surrogate markers need to be examined at the same time during the development stage. Currently, the neutralizing antibody titer in blood is used as an indicator of the expected efficacy of SARS-CoV-2 worldwide. However, it is unclear at this stage how much efficacy can be expected from a vaccine if neutralizing antibodies are induced in the blood. First of all, the approximate effective antibody titer is estimated from serum from immunized animals, animals that have recovered from infection, or serum from patients who have recovered. When considering the development of vaccines for infectious diseases, it is important to understand the pathogenesis of the disease and consider appropriate protective measures. In COVID-19, the target of infection is the epithelium of the upper respiratory tract and alveolar epithelium. Studies on influenza vaccines have shown that neutralizing antibodies in the blood are effective in preventing viral pneumonia in the lungs. Therefore, if we can induce neutralizing antibodies in the blood by the SARS-CoV-2 vaccine, we can expect prevention of viral pneumonia, which has become a problem. While responding to the need for generation of vaccines that are fast-acting, we should aim to develop vaccines that induce mucosal immunity, which is highly likely to suppress infection and prevent epidemics.

## Article Information

### Conflicts of Interest

None

## References

[1] Coronaviridae Study Group of the International Committee on Taxonomy of Viruses, Gorbalenya AE, Baker SC, et al. The species *Severe acute respiratory syndrome-related coronavirus*: classifying 2019-nCoV and naming it SARS-CoV-2. Nat Microbiol. 2020;5(4):536-44. 32123347 10.1038/s41564-020-0695-z PMC7095448

[2] Zhu N, Zhang D, Wang W, et al. A novel coronavirus from patients with pneumonia in China, 2019. N Engl J Med. 2020;382(8):727-33. 31978945 10.1056/NEJMoa2001017 PMC7092803

[3] Roper RL, Rehm KE. SARS vaccines: where are we? Expert Rev Vaccines. 2009;8(7):887-98. 19538115 10.1586/erv.09.43 PMC7105754

[4] Agrawal AS, Tao X, Algaissi A, et al. Immunization with inactivated Middle East Respiratory Syndrome coronavirus vaccine leads to lung immunopathology on challenge with live virus. Hum Vaccin Immunother. 2016;12(9):2351-6. 27269431 10.1080/21645515.2016.1177688 PMC5027702

[5] Houser KV, Broadbent AJ, Gretebeck L, et al. Enhanced inflammation in New Zealand white rabbits when MERS-CoV reinfection occurs in the absence of neutralizing antibody. PLOS Pathog. 2017;13(8):e1006565. 28817732 10.1371/journal.ppat.1006565 PMC5574614

[6] Liu W, Fontanet A, Zhang PH, et al. Two-year prospective study of the humoral immune response of patients with severe acute respiratory syndrome. J Infect Dis. 2006;193(6):792-5. 16479513 10.1086/500469 PMC7109932

[7] Wu LP, Wang NC, Chang YH, et al. Duration of antibody responses after severe acute respiratory syndrome. Emerg Infect Dis. 2007;13(10):1562-4. 18258008 10.3201/eid1310.070576 PMC2851497

[8] Polack FP, Thomas SJ, Kitchin N, et al. Safety and efficacy of the BNT162b2 mRNA Covid-19 Vaccine. N Engl J Med. 2020;383(27):2603-15. 33301246 10.1056/NEJMoa2034577 PMC7745181

[9] Wu F, Zhao S, Yu B, et al. A new coronavirus associated with human respiratory disease in China. Nature. 2020;579(7798):265-9. 32015508 10.1038/s41586-020-2008-3 PMC7094943

[10] Zhou P, Yang XL, Wang XG, et al. A pneumonia outbreak associated with a new coronavirus of probable bat origin. Nature. 2020;579(7798):270-3. 32015507 10.1038/s41586-020-2012-7 PMC7095418

[11] Voysey M, Clemens SAC, Madhi SA, et al. Safety and efficacy of the ChAdOx1 nCoV-19 vaccine (AZD1222) against SARS-CoV-2: an interim analysis of four randomised controlled trials in Brazil, South Africa, and the UK. Lancet. 2021;397(10269):99-111. 33306989 10.1016/S0140-6736(20)32661-1 PMC7723445

[12] Imai M, Iwatsuki-Horimoto K, Hatta M, et al. Syrian hamsters as a small animal model for SARS-CoV-2 infection and countermeasure development. Proc Natl Acad Sci U S A. 2020;117(28):16587-95. 32571934 10.1073/pnas.2009799117 PMC7368255

[13] Iwata-Yoshikawa N, Uda A, Suzuki T, et al. Effects of toll-like receptor stimulation on eosinophilic infiltration in lungs of BALB/c mice immunized with UV-inactivated severe acute respiratory syndrome-related coronavirus vaccine. J Virol. 2014;88(15):8597-614. 24850731 10.1128/JVI.00983-14 PMC4135953

